# “I Like the Idea of It…But Probably Wouldn’t Use It” - Health Care Provider Perspectives on Heart Failure mHealth: Qualitative Study

**DOI:** 10.2196/18101

**Published:** 2020-09-04

**Authors:** Jennifer Dickman Portz, Kelsey Lynett Ford, Kira Elsbernd, Christopher E Knoepke, Kelsey Flint, David B Bekelman, Rebecca S Boxer, Sheana Bull

**Affiliations:** 1 Division of General Internal Medicine University of Colorado School of Medicine Anschutz Medical Campus Aurora, CO United States; 2 mHealth Impact Lab Colorado School of Public Health University of Colorado Anschutz Medical Campus Aurora, CO United States; 3 Division of Cardiology University of Colorado School of Medicine Anschutz Medical Campus Aurora, CO United States; 4 Data Science to Patient Value (D2V) Initiative University of Colorado School of Medicine Anschutz Medical Campus Aurora, CO United States; 5 Adult & Child Consortium for Outcomes Research & Delivery Science University of Colorado School of Medicine Anschutz Medical Campus Aurora, CO United States; 6 Department of Medicine Eastern Colorado Health Care System Department of Veterans Affairs Aurora, CO United States; 7 Institute for Health Research Kaiser Permanente Colorado Aurora, CO United States

**Keywords:** heart failure, information technology, informatics, telemedicine, mHealth

## Abstract

**Background:**

Many mobile health (mHealth) technologies exist for patients with heart failure (HF). However, HF mhealth lacks evidence of efficacy, caregiver involvement, and clinically useful real-time data.

**Objective:**

We aim to capture health care providers’ perceived value of HF mHealth, particularly for pairing patient–caregiver-generated data with clinical intervention to inform the design of future HF mHealth.

**Methods:**

This study is a subanalysis of a larger qualitative study based on interviewing patients with HF, their caregivers, and health care providers. This analysis included interviews with health care providers (N=20), focusing on their perceived usefulness of HF mHealth tools and interventions.

**Results:**

A total of 5 themes emerged: (1) bio-psychosocial-spiritual monitoring, (2) use of sensors, (3) interoperability, (4) data sharing, and (5) usefulness of patient-reported outcomes in practice. Providers remain interested in mHealth technologies for HF patients and their caregivers. However, providers report being unconvinced of the clinical usefulness of robust real-time patient-reported outcomes.

**Conclusions:**

The use of assessments, sensors, and real-time data collection could provide value in patient care. Future research must continually explore how to maximize the utility of mHealth for HF patients, their caregivers, and health care providers.

## Introduction

Nearly 6.5 million Americans have heart failure (HF), which is a leading cause of death, associated with high medical costs and poor quality of life [1]. In all its forms, HF is a chronic condition often characterized by an unpredictable clinical trajectory. HF therapies are complex, including as many as 5 categories of medications when optimized, on top of a variety of possible devices meant to prevent sudden death, improve quality of life and physical functioning, manage syndromes occurring secondary to HF, or some combination of all 3 objectives [2,3]. HF management is thus similarly complex, with alterations to treatment often occurring in response to unsuccessful trials of treatment combinations or hospitalizations, ultimately resulting in the consideration for transplant or mechanical circulatory support [2]. Consistent and ongoing patient-reported data are critical to understanding and predicting clinical decompensation, and methods of capturing such data have historically proven to be elusive [4,5].

The pace of technology continues to drive innovative HF management strategies [6,7]. Consumer-facing mobile technology (eg, wearables, mobile apps, and web-based platforms), known as mHealth (mobile health), offers a modern approach for HF symptom monitoring and psychosocial support. Some of these approaches show promise in improving health care services and health outcomes for patients with HF [8,9]. However, not all off-the-shelf technologies demonstrate evidence of effectiveness or successful adoption [7,10,11]. Despite mixed reviews on their efficacy, enthusiasm for emerging technologies continues among researchers and interventionists [12]. The popularity with real-time interventions, interoperability with electronic health records, and personalization features persist, generating voluminous amounts of data. The clinical usefulness of such robust data in practice remains continually debated [13].

This short paper describes preliminary findings from an ongoing larger mixed-methods research study [14] designed to develop an evidence-based HF mHealth intervention in partnership with all health care stakeholders (ie, patients, caregivers, and providers). The objective of this paper is to illustrate providers’ specific perceived value of HF mHealth, particularly when pairing patient- and caregiver-generated data with meaningful, timely, and effective clinical intervention. Future steps include interviewing and co-designing with patients, health care providers, and family caregivers.

## Methods

This study used a phenomenological [15] design to explore health care providers’ experiences when developing HF mHealth. The qualitative study took place at the University of Colorado Anschutz Medical Campus within the University’s health system—UCHealth. The research team consisted of 1 principal investigator (JDP) and 2 research assistants (KF and KE) experienced in qualitative methods. The study adhered to the Consolidated Criteria for Reporting Qualitative Research (COREQ) [16] and was approved by the Colorado Multiple Institutional Review Board.

### Recruitment and Interview Procedure

Between September 2018 and February 2019, participants were purposefully recruited [17] to partake in semistructured interviews, which were audio-recorded and transcribed. Initially, 10 health care providers with expertise in the treatment of patients with HF were recruited, with snowball sampling methods used to identify an additional 15 providers. Of the 22 that agreed to participate, 20 health care providers from diverse specialties (physicians, nurses, social workers, therapists, and chaplains) participated in interviews. Semistructured interviews were held in a location convenient to the participant and lasted 30-60 minutes.

An 18-question interview guide probed experiences related to the discipline, training, and clinical work with HF. Graphical depictions (ie, “wireframes”) of mobile app elements were created to solicit provider opinions and reactions [18]. Various in-app features included physiologic elements, psychosocial-spiritual assessments, and links to possible resources, beyond standard symptom monitoring. The research team asked about perceived usefulness of mHealth tools for care delivery and care coordination between family caregivers. Participants were incentivized with a US $25 coffee-shop gift card upon completion of the interview.

### Analysis

Two research assistants (KF and KE) read all transcripts and performed double coding procedures. An iterative team-based approach was used to develop a codebook and coding structure based on the research assistants’ epistemological position [19]. The codebook and coding structure were applied to the dataset using Dedoose software (v8.035). Ongoing analysis meetings occurred to validate findings and compare written notes and memos. This consensus-building process ensured the team bracketed their biases and remained reflexive throughout the study. Interrater reliability was calculated for 6 randomly selected transcripts (81% agreement, κ=0.725), reflecting adequate coding consistency. Additionally, during analysis, triangulation occurred to compile resources gathered from interviews (eg, health education materials, mobile app resources, and website suggestions). Until thematic saturation [20] was reached, the research team clustered the codes into categories using significant statements to describe the core essence among participants’ perspectives and selected illustrative quotes reflective of each theme. Member-checking occurred with HF and digital health experts to determine trustworthiness of findings.

## Results

### Participants

The sample included diverse health care provider specialties, including advanced practice nurses (n=7), art/music therapists (n=2), chaplains/spiritual care (n=2), physicians (n=4), registered nurses (n=3), and social workers (n=2). Provider experience ranged from 2 to 17 years (mean 9.78, SD 3.75) working with advanced HF patients. All participants identified as White, 16 identified as female and 4 as male.

### Summary of Significant Statements

A total of 5 thematic clusters resulted from the qualitative analysis. Summarized in Table 1, clusters include bio-psychosocial-spiritual remote monitoring, using sensors and mobile apps, interoperability, data sharing, and useful heuristic preferences.

**Table 1:** Summary of themes with supporting quotes from participant feedback.

Theme	Illustrative quote
Bio-psychosocial-spiritual remote monitoring	*There are a lot of patients, there are a lot of caregivers for whom that stuff is really important. And they do track things and I would imagine it could be really helpful for them going into their doctor to be able to have this information* [1012 – Spiritual Provider] *For me, from a provider perspective, this would help me feel connected. So, if I know the person uses it, likes it, is comfortable with it, and I get immediate messaging about stuff going on, then I can intervene quickly. So, I think from a provider perspective, there’s great comfort in knowing your patient is not heading to crisis. And so, the continual updates are helpful. Balancing that with a lot of unnecessary information.* [1005 – Registered Nurse] *So—because when I see people long-term, we both get to the point where we have trouble remembering what things were like six months ago. …And then you go, well, let’s look. That’s where I use data. Let’s look two weeks ago. Two weeks ago, you were saying things were good. So, it hasn’t been bad for six months. You were kind of good two weeks ago but now we’re having a blip. And that will encourage—that will give two things. That will give people perspective on their disease, but it will also be huge for prognosis for a provider.* [1005 – Registered Nurse] *I think symptom tracking is good because a lot of times whether you're the patient or the caregiver, both want to know what symptoms to look out for to be causes of concern like when do I need to call my doctor, when do I need to get to an emergency room.* [1016 – Social Worker]
Sensor technologies and potential of real-time data	*If that’s one less thing the person has to enter, then I’m all for it.* [1005 – Registered Nurse] …*for the most part I think that it’s really good because it empowers the patient to take control of their self.* [1019 – Advanced Practice Nurse] *So if you had somebody that had really high heart rates and said they didn’t feel well and that’s for a step like who’s going to read that data, who’s going to get them in and is it the patient’s responsibility to still call and say, ‘Hey, I feel terrible.’* [1011 – Advanced Practice Nurse]
Interoperability for a personalized experience	…*[if] they had a desire for [seeing a spiritual provider or social worker]; for there to actually be a vehicle where they're not dependent on someone asking or them to think to ask for it. Maybe they didn't even know they could.* [1012 – Spiritual Provider] *So yeah, I don’t necessarily have to have my diet fitness app integrated with my heart failure app, per se. I’m not sure there’s huge advantage to that, unless you're linking them somehow. So OK; so now I’m figuring how much sodium there is and then my sodium on my fitness diet app is looking at my weight and saying, “Well as it turns out, you say your weight’s going up and your sodium consumption over the last four days has been 4,000 milligrams a day, which is more,” and then you're like giving real-time feedback to the patient about how, potentially, what they’re doing in terms of their health behaviors is affecting their objective measures. That would be nice. That seems pretty complex, though.”* [1018 – Physician]
Tailored assessment and sharing of data	*So, I see how it could be helpful for sure. But I just see the patient as potentially having some problems with it. I see the caregiver as going oh, my gosh, aren’t I doing enough? And so, there can be guilt that this could trigger. Used with the right people—and if you had the ability to (tailor) opt in or opt out of this feature, that’s how you might be able to solve that. Some people would love it. I know they would. But I just don’t have a good feel for the percentages on that and so maybe you just opt in or opt out.* [1005 – Registered Nurse] *I think that’s an individual thing. Some caregivers, family members want to know everything like this and some of them only want to know if something bad is going on. So, I guess that would be a decision between the patient and the caregiver about what they wanted to do from that*. [1011 – Advanced Practice Nurse]
Usefulness of patient-reported outcomes in practice	…*in clinic now when I try to get a point across to the patients, I’ll graph some of the data and you can see it taking off. And it’s really—I don’t know if it’s motivating to them, but they’re real interested in those trends.* [1020 – Registered Nurse] *I think the way this could be useful is just looking at trends. You know, if you were able to say well on Monday this is how I felt and then on Thursday of that week, you don’t really remember what you put for Monday and if you could track some sort of bar graph or something for your responses, all of a sudden you click on a summary and it’s like oh, I’ve been a little tired for the last 12 days and I’ve been coughing and—or whatever it is.* [1000 – Registered Nurse] …*the problem is you have so many different users. You have users with visual impairment who listen to their smartphone through their ear… It'd be cool if you could design the app where it’s sort of tailored to different disabilities like see this for visually impaired or see this for hearing…* [1015 – Physician]

### Bio-Psychosocial-Spiritual Remote Monitoring

Many health care providers, especially clinical providers (physicians and nurses), mentioned that some of their patients already tracked HF symptoms in a variety of ways, most commonly handwritten journals. In addition, several providers discussed the value of this information for caregivers and the utility of a mobile app for consolidating patient-reported monitoring metrics, thus helping family members and caregivers feel updated on their loved one’s condition. Psychosocial assessments and questions about practical help, while less interesting to clinical providers, were recognized as a way to get a more well-rounded picture of patients with complex chronic illness. However, a few participants raised concerns about the utility of psychosocial assessment in a digital platform. Concerns included the ability of an app to link patients to reliable follow-up resources based on responses.

### Sensor Technologies and Potential of Real-Time Data

Most providers were excited about sensor technology such as weight tracking, home blood pressure monitoring, and physical activity monitoring and its utility in minimizing patient-driven data collection. A few providers mentioned technical difficulties (eg, wireless access, end-user error) or practical challenges (eg, ability to fit sensor-generated data monitoring into the clinical workflow) in past experiences with sensor technology. While most providers agree that this type of information is useful during patient encounters, the ability to monitor the data was a concern. The technology allows an abundance of data to be collected 24 hours a day, 7 days a week via sensors with no associated plan as to how to monitor that data; this was consistently raised across provider specialties.

### Interoperability for a Personalized Experience

Many providers discussed the potential to sync patient-generated data with the electronic health record and patient portal. However, many providers raised concerns about various limitations to make this information useful to the care team and caregivers. For example, a few providers reported that there are many symptom-tracking technologies, which collect patient-reported outcomes, currently linked to the electronic health record but are rarely monitored on the clinical side.

Beyond clinical interoperability, providers discussed the ability of HF mHealth to interface with other apps that provide psychosocial support, reporting it to be useful to sync with other commonly used apps (eg, mindfulness apps, music apps, Google calendars, and shared-list apps). Most providers recommended this would be more helpful than having an additional modality to find resources. Many providers discussed the many existing HF resources and raised the need for an app assessment to link to actionable resources (eg, ask patient about medical power of attorney and provide a web link to fulfill the need; provide functionality to request a spiritual provider or social worker based on remote monitoring responses). Such personalization appeared advanced to providers. However, most of them reported how interoperability would improve usability.

### Tailored Assessment and Sharing of Data

Many providers reported reliance on caregivers during patient encounters as an additional perspective of patient health status and as a secondary source when discussing care plans. Thus, most providers agreed that caregivers having access to patient data would be helpful. However, concerns were raised around patient privacy and caregiver fatigue or guilt. Furthermore, providers discussed varying preferences for specific caregivers; and therefore, tailoring data-sharing options would be critical.

### Usefulness of Patient-Reported Outcomes in Practice

Providers almost unanimously preferred graphs showing trends in HF symptoms and physiologic measures. Many providers were concerned with facilitating data collection in a patient-centered, noninvasive way that does not collect more data than used. Suggestions to improve accessibility, language, and literacy of assessment questions remained a key consideration. In addition, several providers brought up disabilities such as diminished eyesight or hearing among an older population and discussed advanced technology features such as voice activation integrated into an app to increase accessibility of mobile apps in this population.

## Discussion

This study reports provider experiences and opinions regarding the development of HF mHealth that will maximize patient, family, and provider clinical utility. Our findings suggest providers remain interested in various innovative solutions for HF patients and their caregivers. The use of assessments, sensors, and real-time data collection could provide value in patient care. However, providers remained skeptical of the clinical usefulness of vast data and real-time patient reported outcomes [7,21].

Although HF mHealth is increasing in popularity, concerns with privacy, confidentiality, and overburden of electronic medical record alerts with interoperable technologies may only complicate the clinical practice [22,23]. This contradicts current endorsements of real-time data generation in mHealth (eg, just-in-time adaptive interventions and ecological momentary interventions) to inform clinical decision making [24]. Instead, we found that health care providers “…like the idea of it but, personally, probably wouldn’t use it.”

In conclusion, future HF mHealth research must consider its usefulness in practice for patients, caregivers, and health care providers. Although innovative mHealth technologies offer promise in improving HF outcomes and quality of life for patients, the interventions and tools must remain relevant and useful without causing an additional burden for the patient, caregiver, and care team. With the increasing adoption of HF mHealth, understanding multiple perspectives remains critical for sustained engagement, thus improving the impact of HF mHealth on patients, families, society, and the health care system.

## Abbreviations

COREQ: Consolidated Criteria for Reporting Qualitative Research
HF: heart failure
mHealth: mobile health

## References

[1] Benjamin Emelia J, Muntner Paul, Alonso Alvaro, Bittencourt MS, Callaway CW, Carson AP, Chamberlain AM, Chang AR, Cheng S, Das Sandeep R, Delling Francesca N, Djousse Luc, Elkind Mitchell S V, Ferguson Jane F, Fornage Myriam, Jordan Lori Chaffin, Khan Sadiya S, Kissela Brett M, Knutson Kristen L, Kwan Tak W, Lackland Daniel T, Lewis Tené T, Lichtman Judith H, Longenecker Chris T, Loop Matthew Shane, Lutsey Pamela L, Martin Seth S, Matsushita Kunihiro, Moran Andrew E, Mussolino Michael E, O'Flaherty Martin, Pandey Ambarish, Perak Amanda M, Rosamond Wayne D, Roth Gregory A, Sampson Uchechukwu K A, Satou Gary M, Schroeder Emily B, Shah Svati H, Spartano Nicole L, Stokes Andrew, Tirschwell David L, Tsao Connie W, Turakhia Mintu P, VanWagner Lisa B, Wilkins John T, Wong Sally S, Virani Salim S, American Heart Association Council on EpidemiologyPrevention Statistics CommitteeStroke Statistics Subcommittee (2019). Heart Disease and Stroke Statistics-2019 Update: A Report From the American Heart Association. Circulation.

[2] Yancy CW, Jessup M, Bozkurt B, Butler J, Casey DE, Colvin MM, Drazner MH, Filippatos GS, Fonarow GC, Givertz MM, Hollenberg SM, Lindenfeld J, Masoudi FA, McBride PE, Peterson PN, Stevenson LW, Westlake C (2017). 2017 ACC/AHA/HFSA Focused Update of the 2013 ACCF/AHA Guideline for the Management of Heart Failure: A Report of the American College of Cardiology/American Heart Association Task Force on Clinical Practice Guidelines and the Heart Failure Society of America. J Card Fail.

[3] Yancy CW, Januzzi JL, Allen LA, Butler J, Davis LL, Fonarow GC, Ibrahim NE, Jessup M, Lindenfeld J, Maddox TM, Masoudi FA, Motiwala SR, Patterson JH, Walsh MN, Wasserman A (2018). 2017 ACC Expert Consensus Decision Pathway for Optimization of Heart Failure Treatment: Answers to 10 Pivotal Issues About Heart Failure With Reduced Ejection Fraction: A Report of the American College of Cardiology Task Force on Expert Consensus Decision Pathways. J Am Coll Cardiol.

[4] Masterson Creber RM, Maurer MS, Reading M, Hiraldo G, Hickey KT, Iribarren S (2016). Review and Analysis of Existing Mobile Phone Apps to Support Heart Failure Symptom Monitoring and Self-Care Management Using the Mobile Application Rating Scale (MARS). JMIR Mhealth Uhealth.

[5] Lee S, Riegel B (2018). State of the Science in Heart Failure Symptom Perception Research: An Integrative Review. J Cardiovasc Nurs.

[6] Bashi N, Karunanithi M, Fatehi F, Ding H, Walters D (2017). Remote Monitoring of Patients With Heart Failure: An Overview of Systematic Reviews. J Med Internet Res.

[7] Athilingam P, Jenkins B (2018). Mobile Phone Apps to Support Heart Failure Self-Care Management: Integrative Review. JMIR Cardio.

[8] Riegel B, Moser DK, Buck HG, Dickson VV, Dunbar SB, Lee CS, Lennie TA, Lindenfeld J, Mitchell JE, Treat-Jacobson DJ, Webber DE, American Heart Association Council on CardiovascularStroke Nursing; Council on Peripheral Vascular Disease;Council on Quality of CareOutcomes Research (2017). Self-Care for the Prevention and Management of Cardiovascular Disease and Stroke: A Scientific Statement for Healthcare Professionals From the American Heart Association. J Am Heart Assoc.

[9] Carbo A, Gupta M, Tamariz L, Palacio A, Levis S, Nemeth Z, Dang S (2018). Mobile Technologies for Managing Heart Failure: A Systematic Review and Meta-analysis. Telemed J E Health.

[10] Panjrath G, Bostrom L, Witkin L, Katz R (2018). Moving Mobile Health Forward: Combining mHealth with Community Health Workers Bolsters Heart Failure Care. Journal of Cardiac Failure.

[11] Marcolino MS, Oliveira JAQ, D'Agostino M, Ribeiro AL, Alkmim MBM, Novillo-Ortiz D (2018). The Impact of mHealth Interventions: Systematic Review of Systematic Reviews. JMIR Mhealth Uhealth.

[12] Dickinson MG, Allen LA, Albert NA, DiSalvo T, Ewald GA, Vest AR, Whellan DJ, Zile MR, Givertz MM (2018). Remote Monitoring of Patients With Heart Failure: A White Paper From the Heart Failure Society of America Scientific Statements Committee. J Card Fail.

[13] Snyder CF, Aaronson NK (2009). Use of patient-reported outcomes in clinical practice. Lancet.

[14] Dickman Portz J, Ford K, Bekelman DB, Boxer RS, Kutner JS, Czaja S, Elsbernd K, Bull S (2020). "We're Taking Something So Human and Trying to Digitize": Provider Recommendations for mHealth in Palliative Care. J Palliat Med.

[15] Starks H, Trinidad Susan Brown (2007). Choose your method: a comparison of phenomenology, discourse analysis, and grounded theory. Qual Health Res.

[16] Tong A, Sainsbury P, Craig J (2007). Consolidated criteria for reporting qualitative research (COREQ): a 32-item checklist for interviews and focus groups. Int J Qual Health Care.

[17] Creswell J, Poth C (2018). Qualitative inquiry & research design: Choosing among five approaches.

[18] Brhel M, Meth H, Maedche A, Werder K (2015). Exploring principles of user-centered agile software development: A literature review. Information and Software Technology.

[19] Carter SM, Little M (2007). Justifying knowledge, justifying method, taking action: epistemologies, methodologies, and methods in qualitative research. Qual Health Res.

[20] Ando H, Cousins R, Young C (2014). Achieving Saturation in Thematic Analysis: Development and Refinement of a Codebook. Comprehensive Psychology.

[21] Seto E, Leonard KJ, Masino C, Cafazzo JA, Barnsley J, Ross HJ (2010). Attitudes of heart failure patients and health care providers towards mobile phone-based remote monitoring. J Med Internet Res.

[22] Sarwar CM, Vaduganathan M, Anker SD, Coiro S, Papadimitriou L, Saltz J, Schoenfeld ER, Clark RL, Dinh W, Kramer F, Gheorghiade M, Fonarow GC, Butler J (2018). Mobile health applications in cardiovascular research. Int J Cardiol.

[23] Turakhia MP, Desai SA, Harrington RA (2016). The Outlook of Digital Health for Cardiovascular Medicine: Challenges but Also Extraordinary Opportunities. JAMA Cardiol.

[24] Dolley S (2018). Big Data's Role in Precision Public Health. Front Public Health.

